# Mechanism of azithromycin inhibition of HSL synthesis in *Pseudomonas aeruginosa*

**DOI:** 10.1038/srep24299

**Published:** 2016-04-14

**Authors:** Jianming Zeng, Ni Zhang, Bin Huang, Renxin Cai, Binning Wu, Shunmei E, Chengcai Fang, Cha Chen

**Affiliations:** 1 Dept. of Laboratory Medicine, Guangdong Provincial Hospital of Traditional Chinese Medicine, Guangzhou Higher Education Mega Center, 55 Neihuan Xi Road, Panyu District, Guangzhou 510006, China; 2 Clinical Microbiology Laboratory, Guangdong Academy of Medical Sciences & Guangdong General Hospital, Guangzhou 510080, China; 3 Dept. of Laboratory Medicine, the First Affiliated Hospital of Sun Yat-sen University, 58 Zhongshan Er Road, Guangzhou 510080, China

## Abstract

*Pseudomonas aeruginosa* is an opportunistic pathogen and a leading cause of nosocomial infections. Unfortunately, *P. aeruginosa* has low antibiotic susceptibility due to several chromosomally encoded antibiotic resistance genes. Hence, we carried out mechanistic studies to determine how azithromycin affects quorum sensing and virulence in *P. aeruginosa*. *lasI* and *rhlI* single and double mutants were constructed. We then undertook a quantitative approach to determine the optimal concentration of azithromycin and culture time that can affect the expression of HSLs. Furthermore, based on the above results, the effect on quorum sensing was analyzed at a transcriptional level. It was found that 2 μg/mL azithromycin caused a 79% decrease in 3-oxo-C12-HSL secretion during cultivation, while C4-HSL secretion was strongly repressed in the early stages. Azithromycin acts on ribosomes; to determine whether this can elicit alternative modes of gene expression, transcriptional regulation of representative virulence genes was analyzed. We propose a new relationship for *lasI* and *rhlI*: *lasI* acts as a cell density sensor, and *rhlI* functions as a fine-tuning mechanism for coordination between different quorum sensing systems.

Quorum sensing (QS) is an important global regulatory mechanism in bacteria that enables individual bacteria to coordinate their behavior in response to cell density^1^. The QS system relies on self-generated signaling molecules to coordinate gene expression^2^,^3^. Two QS systems, i.e., the las and rhl systems, have been identified in *P. aeruginosa* (*PA*). In the las QS system, the *lasI* gene product directs the formation of a diffusible extracellular signal, N-(3-oxododecanoyl)-L-HSL (3-oxo-C12-HSL or OdDHL), which interacts with LasR to activate a number of virulence genes. On the other hand, the *rhlI* gene product catalyzes the synthesis N-butyryl-L-HSL (C4-HSL or DHL)^4^,^5^. This diffusible signal, in conjunction with RhlR, activates the expression of other virulence genes. Although the two QS systems have distinct downstream targets, they are still hierarchically connected^6^. It has been found that the las system positively regulates the expression of both rhlR and rhlI^7^.

Approximately 600 genes are regulated by QS in *PA*, and most of the regulation occurs during the stationary phase of growth^8^. The Las system mainly regulates toxins (exotoxin A and exoenzymes) and proteases (elastase, LasA protease and alkaline protease), and hemolysins (phospholipase and rhamnolipid) are regulated by the rhl system^9^,^10^. 4-hydroxy-2-alkylquinoline (HAQ), and acyl homoserine lactone (AHL) are two well-characterized signaling molecules^11^. HAQ is involved in cell-to-cell communication pathway, while AHL is used to regulate growth and gene expression. The regulation of gene expression allows the integration of cell density with virulence factor production. Virulence is regulated by both external factors and interaction with the QS systems. Previous studies have reported that sub-inhibitory concentrations of antibiotics not only inhibit QS but also act as signaling molecules for triggering virulence factor production^12^,^13^.

*PA* is a opportunistic human pathogen and a leading cause of nosocomial infections, particularly in immunocompromised patients, including those with cancer, burns and cystic fibrosis^14^,^15^,^16^. The emergence and rapid spread of multidrug-resistant *PA* (MDRPA) isolates that cause serious nosocomial infections are of great concern^17^,^18^. The extensive and irrational use of antimicrobial agents has promoted resistance in *PA*. There are now a number of multidrug-resistant strains, which make clinical treatment difficult^19^. The resistance mechanisms of *PA* are very complex; horizontal gene transfer (HGT) is an important mechanism^20^. Azithromycin (AZM), a member of the macrolide class of antibiotics, is used to treat certain bacterial infections, which are primarily caused by gram-positive bacteria but also some gram-negative pathogens. Many clinical and experimental studies have shown the beneficial effects of AZM in patients with diffuse panbronchiolitis and cystic fibrosis, which are associated with *PA* infection^21^,^22^,^23^.

A study by Henkel^24^ showed that AZM has the potential to inhibit QS signal molecules and attenuate the virulence of *PA*. Sub-MIC concentrations of AZM were found to inhibit the production of QS signals, swimming, swarming and twitching motilities, and biofilm formation *in vitro*. AZM affects QS by interfering with one of the following pathways: biofilm formation^25^, translation due to its interaction with ribosomes^26^, or increase in outer membrane permeability^27^. Moreover, 2 μg/mL AZM (1/64th of the MIC) showed promising results on QS-dependent virulence factor production, biofilm formation, and oxidative stress resistance in *PA*^28^,^29^.

The mechanisms of QS regulation have mostly been described qualitatively. However, the time course of HSL synthesis and the peak activity of this process are currently unclear, and a quantitative analysis of AZM-mediated inhibition of QS signals has yet to be performed. In this study, we applied HPLC-MS to assay both C4-HSL and 3-oxo-C12-HSL synthesis and the effect of the *lasI* and *rhlI* genes in both *lasI* and *rhlI* mutants and wild-type PAO1 following treatment with AZM. Additionally, the effect of AZM on the transcription of toxin, elastase and hemolysin genes was investigated to assess the transcriptional dysfunction due to reduced LasI. We also assessed the expression of the HAQ’s downstream effector genes (*phnAB*) after adding AZM.

## Materials and Methods

### Construction of *P. aeruginosa* PAO1 *lasI* and *rhlI* mutant strains

#### Bacterial strains, plasmids, and media

The bacterial strains and plasmids used in this study are shown in Table 1. PAO1 was a gift from Dr. Zhou Lin (Children’s Hospital of Chongqing Medical University). Strains were routinely grown in rich liquid or solid (15 g/L agar) Luria-Bertani medium (LB). The medium was supplemented with ampicillin (Amp, 60 μg/mL), chloramphenicol (C, 34 μg/mL) or gentamicin (GM, 30 μg/mL). Solid LB medium with 10% sucrose and without NaCl was used to select for plasmid excision from the chromosome in the gene allelic exchange experiments. Omission of NaCl from this medium was previously shown to improve sucrose counter-selection in *Escherichia coli* (*E. coli*).

#### DNA techniques

The rTaq DNA polymerase, restriction endonucleases, DNA-modifying enzymes, Klenow fragment, DNA kination kit and DNA ligation kit used in this study were purchased from TaKaRa. The plasmid extraction kit and DNA gel purification kit were purchased from Guangdong Dongsheng Biotech Corporation. Antarctic phosphatase and T4 DNA ligase were purchased from NEB.

The PCR-amplified *lasI* gene upstream fragment A (*lasI*-P4: AATTCTATGAGTCGCTGCCGACTCTGG CGGTTTCATGACGGGGAC, *lasI*-P2: CGGTCGCCTATCTCGGTATCA) from PAO1 was ligated into pMD18T by TA cloning to construct pMD18B, and the PCR-amplified *lasI* gene downstream fragment (*lasI*-P1: TAC*AAGCTT*AAGAAGAACGTAGCGCTATGGC, *lasI*-P3: CTC*AAGCTT*CACTTCCTCC AAATAGGAA, containing a HindIII site) was cloned into the HindIII site of pMD18B to create pMD18AB.

Two primers (*rhlI*-P1: TCC*CCCGGG*TTATCGATCAGGGCTTACTGCAATGA, *rhlI*-P3: TCC*CCCGGG*AGCATGACCAAGTCCCCGTGTCG, containing a SmaI site) were synthesized to amplify fragment C, and C was then digested with SmaI and ligated into pUC19C. Fragment D was amplified with primers (*rhlI*-P4: AATTCTATGAGTCGCTGCCGACTCTGGCGGTTTCATGACGGGGAC, *rhlI*-P2: GG*GGTACC*AGAACGCCCAGAGGACGGTGAC, containing a KpnI site). The pUC19C vector and D were digested with KpnI, followed by ligation to obtain pUC19CD.

The pGSM-ΔlasI vector was derived from pMD18AB and pCVD442 in several steps. First, the GM sequence was amplified from the pBBR-LuxAB plasmid and was TA cloned into pMD18-GM, and fragments of sacB and mob from pCVD442 were amplified^30^. The GM fragment was phosphorylated using the DNA kination kit and inserted into a SmaI site on pMD18AB to create pG-ΔlasI, and mob was TA cloned into pMD18-mob.

For the construction of pGSM-ΔlasI, a 1.8 kb PCR fragment of sacB was ligated into the XbaI site of pG-ΔlasI to form pGS-ΔlasI, and the mob sequence of a 2.2 kb SmaI/HindIII fragment from pMD18-mob was blunt-ended and ligated into pGS-ΔlasI to construct pGSM-ΔlasI.

An 831 bp XbaI/HindIII fragment from pMD18-GM was sub-cloned into pUC19CD, forming pG-ΔrhlI. For the construction of pGSM-ΔlasI, a 1.8 kb PCR fragment of sacB was ligated into a HindIII site of pG-ΔrhlI, yielding pGS-ΔrhlI, and the mob sequence of a 2.2 kb SmaI/HindIII fragment from pMD18-mob was blunt-ended and ligated into pGS-ΔrhlI to construct pGSM-ΔrhlI.

Plasmids pGSM-ΔlasI and pGSM-ΔrhlI were mobilized by conjugation from *E. coli* SM10λpir into *P. aeruginosa* PAO1 to generate a lasI mutant strain (PA-ΔlasI) and an rhlI mutant strain (PA-rhlI), respectively. For the generation of an rhlI and lasI double mutant (PA-ΔlasIrhlI), pGSM-ΔlasI was conjugally transferred from *E. coli* SM10λpir into a *P. aeruginosa* PA-ΔrhlI mutation strain.

#### MIC determination

The MIC of AZM (Pfizer, Germany) was determined according to the CLSI (2012)^31^ guidelines with the standard *P. aeruginosa* strain PAO1 and the mutant strains PA-ΔlasI, PA-Δrhl, and PA-ΔlasIrhlI. Briefly, from stock solutions of AZM (10 mg/mL), different dilutions (5–200 mg/mL) were prepared. The minimal concentration of antibiotic resulting in no visible growth was taken as the MIC after 6 h of culture. For all further experiments, 1/64th of the MIC of AZM was used for all four strains^32^.

### HSL analysis by HPLC-MS/MS

#### Chemicals and standards

The standards N-butyryl-L-HSL (C4-HSL) and N-(3-oxododecanoyl)-L-HSL (3-oxo-C12-HSL) were purchased from Sigma. Methanol, acetonitrile (HPLC grade), and formic acid (MS grade) were purchased from Fisher Scientific (Loughborough, UK). Acetic acid and ethyl acetate (HPLC grade) were obtained from Guangzhou Chemical Reagent Factory (Guangzhou, China), and ultra-pure water (>18 MΩ/cm) was obtained from a Milli-Q water Elga Maxima water purification system (Merck KgaA, Germany).

#### Growth curve

PAO1, PA-ΔrhlI and PA-ΔlasIrhlI were grown in 3 mL of LB medium at 37 °C with shaking. The cultures were simultaneously supplemented with AZM at a final concentration of 2 μg/mL. The cultures were collected after 0, 2, 4, 6, 8, 10, or 12 h of growth and assayed at OD_600_.

#### Quantification of HSLs by HPLC-MS/MS

After clarification by centrifugation, 1 mL of the cell-free supernatant was extracted three times with an equal volume of ethyl acetate, which was supplemented with 0.2 M acetic acid^33^. Thereafter, the combined organic phase was dried in an N2 stream, redissolved in 1 mL of methanol and stored at −20 °C to prepare other stock solutions. Twenty microliters of each of these samples was added to 980 ìL of methanol for a 50-fold dilution immediately prior to HPLC-MS/MS analysis. All samples were filtered through 0.22 μm nylon disk filters before HPLC-MS/MS analysis^34^.

The HPLC-separated compounds were detected by electrospray ionization ion trap mass spectrometry (ESI-MS) using a Bruker Esquire-LC spectrometer (Bruker Daltonic, Germany) under positive-ion conditions. The analysis of two structurally distinct HSLs requires fast and selective analysis. The choice of + ESI mode was based upon the greater sensitivity for both HSL analytes; this was relatively straightforward because all the analytes demonstrated protonated [M + H]+ species as the dominant pseudo-molecular ion.

Ten microliters of the sample was injected for HPLC-MS/MS analysis and introduced onto a ZORBAX SB-C18 column (4.6 × 100 mm, 2.1 μm). The HPLC system used 0.3% formic acid in water as mobile phase A, and mobile phase B was 50% acetonitrile in methanol. The gradient profile was as follows: isocratic for 3 min with 10% B, then a further gradient with 90% B over 2 min, followed by 10% B for 5 min, at a flow rate of 0.5 mL/min. All mass spectrometry (MS) experiments were conducted on a 4000 QTRAP hybrid triple-quadrupole linear ion trap mass spectrometer (Applied Biosystems, Foster City, CA, USA) equipped with a TurboIon source used in positive ion electrospray mode. A Windows XP (Microsoft, Redmond, WA, USA) workstation running Analyst (version 1.6) was used for data acquisition and processing.

The working flow rate and gas were as follows: curtain, gas 1 and 2 were nitrogen, 60 L/h, and 60 L/h, respectively. The ion source potential was 5,500 Vm, and the source was held at 550 °C. Quantification was performed using Analyst 1.6 in Quantitate mode.

Six-point standard curves were generated for C4-HSL and 3-oxo-C12-HSL, and the standard mixture was reanalyzed after every sixth sample.

#### Recovery Test

The recovery of analytes from the medium was determined by adding a standard mixture of C4-HSL and 3-oxoC12-HSL at low (10 μg/mL) and high (100 μg/mL) concentrations to 3 mL of LB culture media. Each sample was extracted three times with an equal volume of ethyl acetate. The recovery was calculated by comparing the response ratios of spiked extracted medium with a standard mixture prior to extraction.

Precision was calculated from the relative standard deviation (RSD) of the replicates (n = 5), and accuracy was calculated by direct comparison of mean measured levels of spiked analytes with expected concentrations for unextracted standards.

### Real-time quantitative RT-PCR

For real-time quantitative RT-PCR (qPCR) analyses, total RNA from the indicated cells was extracted using the total RNA isolation reagent (Promega, Madison, WI, USA). Reverse transcription was performed with the PrimeScript RT reagent kit (TaKaRa, Dalian, Liaoning, China) using 1 ìg of total RNA. SYBR Green qPCR Master Mixes (ThermoFisher Scientific, Waltham, MA, USA) were used for qPCR detection of the cDNA, and qPCR reactions were performed on ViiA^TM^ 7 Dx system (Applied Biosystems, Foster, CA, USA). The level of target genes was normalized to the expression of an internal control gene (rpoD), which yielded a 2^−ΔΔCt^ value. Sequences for primers are listed in Appendix 1.

### Statistical analysis

All experiments were carried out in triplicate to validate the reproducibility of the experiments. The results were analyzed statistically using repeated measures analysis of variance with SAS 9.2 software to calculate *P* values. *P* < 0.05 was taken as statistically significant.

## Results

The AZM MIC for *P. aeruginosa* PAO1 was determined to be 128 mg/mL, the same as described previously. Therefore, 2 μg/mL AZM (1/64th of the MIC) was added to all four strains in these experiments.

### HPLC-MS/MS Analysis

Using the experimental conditions described in the Materials and Methods section, the analogues of C4-HSL and 3-oxo-C12-HSL were separated. Active fractions were located in two single peaks (C4-HSL and 3-oxo-C12-HSL) with retention times of 5.09 min and 7.0 min, respectively. Two compounds with these retention times were obtained from *P. aeruginosa* PAO1 supernatants but were absent from PA-ΔlasIrhlI supernatants.

The MS parameters (precursor and product ions used for MRM transitions and corresponding optimized voltages) are listed in Table 2, which shows that common product ion fragments could be used for individual families of analytes, i.e., m/z 102 for HSLs. Our detection limits were 0.05 ng/mL and 50 ng/mL.

Extraction of the supernatants was performed as described in previously, and detection and quantification of N-acylhomoserine lactones was performed by HPLC-MS/MS.

#### Recovery Test

The bacterial culture media potentially containing HSLs are complex matrices containing components of growth media and bacterial exoproducts, which could affect the specificity and sensitivity of HPLC-MS/MS detection. To examine the influence of the medium on the extraction, we added a standard mixture of HSLs to the culture media at either low (10 μg/mL) or high (100 μg/mL) concentrations for extraction.

For the low concentration samples, the yields of C4-HSL and 3-oxo-C12-HSL were approximately 64.4% and 97.6%, respectively, while for the high concentration samples, the yields of C4-HSL and 3-oxo-C12-HSL were approximately 86.8% and 89.1%, respectively (Table 3).

Precision was calculated from the relative standard deviation (RSD) of the replicates (n = 5), and accuracy was calculated by direct comparison of mean measured levels of spiked analytes with expected concentrations for unextracted standards.

### Effect of mutation and AZM on *P. aeruginosa* growth

**Figure 1:**
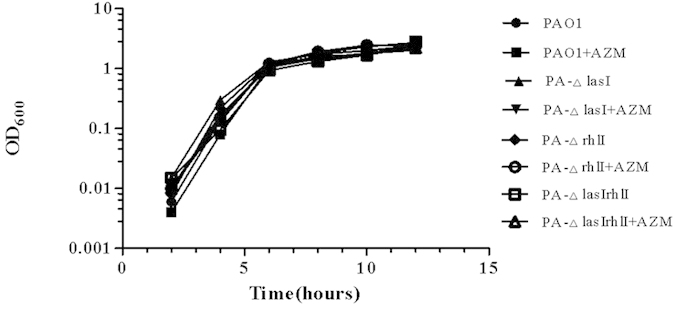
Growth curves of PA-Δ lasI, PA-△ rhlI, PA-△ lasIrhlI and their parent strain PAO1. The relationship of OD_600_ to viable count was equivalent for all strains examined. Each point indicates the mean of the OD_600_ values. AZM indicates this strain was treated with 2 μg/mL of azithromycin.

As shown in Fig. 1, bacterial growth curves indicated that there were no differences between PAO1 and the mutants deficient in *lasI*, *rhlI* and *lasI*/*rhlI* in terms of growth at 0, 2, 4, 6, 8, 10 and 12 h. Based on the data, 0–8 h is the exponential phase, followed by the early stationary phase. Growth comparisons to PAO1 were performed using paired t-test analyses (*P* > 0.05). The results indicate that QS mutations, i.e., PAO1 deficient in *lasI*, *rhlI* and *lasI*/*rhlI*, had almost no influence on *P. aeruginosa* growth. Moreover, 2 μg/mL of AZM did not affect the growth of the strains compared with untreated controls (*P* > 0.05) (Fig. 1).

### Effect of *lasI* and *rhlI* mutations on QS signals

**Figure 2:**
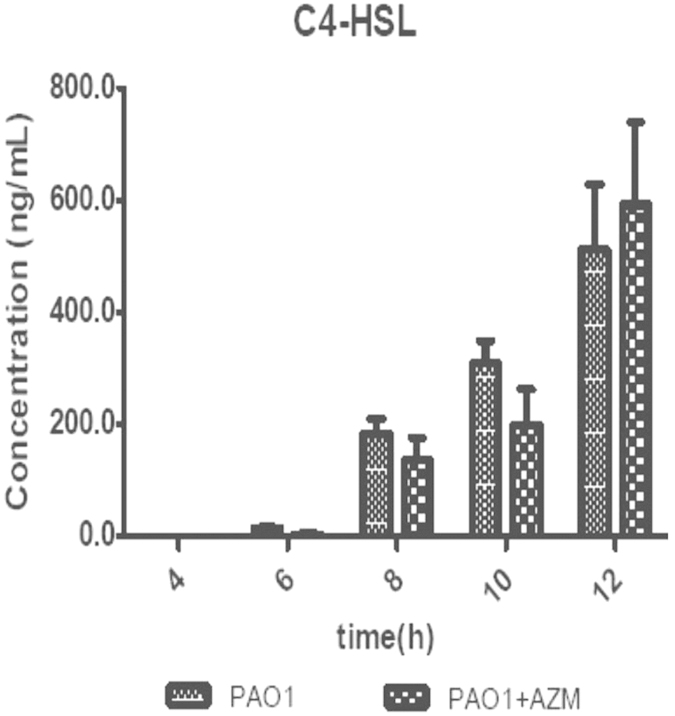
C4-HSL secretion in wild-type (PAO1) compared with mutant strains. Because PA-Δ lasI, PA-Δ rhlI and PA-Δ lasIrhlI did not produce C4-HSL, the data are not shown. +AZM indicates strains treated with azithromycin (2 μg/mL). PAO1 treated with 2 μg/mL AZM showed no statistically significant differences compared to the untreated control. The results are presented as the mean ± SD obtained from three independent experiments.

**Figure 3:**
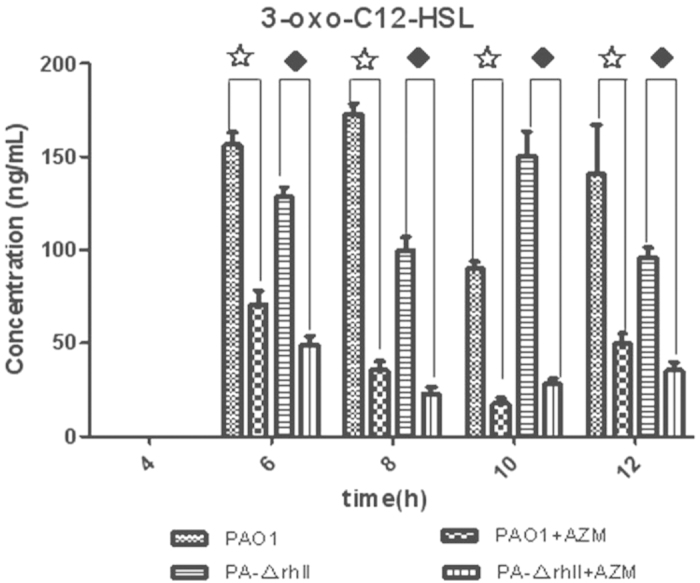
3-Oxo-C12-HSL secretion in wild-type (PAO1) compared with mutant strains. Because PA-Δ lasI and PA-Δ rhlI did not produce 3-oxo-C12-HSL, the data are not shown.+AZM indicates strains treated with azithromycin (2 μg/mL). The results are presented as the mean ± SD obtained from three independent experiments. ⋆ denotes PAO1 compared with 2 μg/mL AZM treatment, with statistically significant differences. ♦ denotes PA-Δ rhlI compared with 2 μg/mL AZM treatment, with statistically significant differences.

No C4-HSLs were detected in *P. aeruginosa* PAO1 in the early growth phase, which was between 2–4 h (Fig. 2). However, 3-oxo-C12-HSL was detected at 6 h, during the exponential phase of growth, and secretion was maintained until 12 h; the concentration ranged from 89.6 ng/mL to 172.8 ng/mL (Fig. 3). A comparison of the different time points for the secretion of 3-oxo-C12-HSL showed no statistically significant differences, *P* > 0.05. The secretion of C4-HSL increased with time, with a maximum concentration of 512.2 ng/mL, which was stable from 10 h to 12 h, i.e., in the stationary phase. (A comparison of the different time points for the secretion of C4-HSL showed statistically significant differences, *P* < 0.05.) For PA-ΔrhlI, C4-HSL was not detected, but 3-oxo-C12-HSL production was similar to that of PAO1; there was no significant difference between the two strains (*P* > 0.05). For PA-ΔlasI, 3-oxo-C12-HSL was not detected, and C4-HSL levels were below the detection limits. For PA-ΔlasIrhlI, there was no C4-HSL or 3-oxo-C12-HSL secretion. In the QS system, the lasI gene product directs the formation of the diffusible extracellular signal 3-oxo-C12-HSL, and the rhlI product catalyzes the synthesis of C4-HSL^35^; therefore, the las system positively regulates the expression of rhlI^36^.

### Effect of AZM on QS signals

At 6 h, 8 h, and 10 h, C4-HSL levels in the PAO1 strain were 13.1 ng/mL, 183.2 ng/mL, and 308.4 ng/mL, respectively. With addition of AZM, the levels in the PAO1 strain decreased to 3.2 ng/mL, 119.7 ng/mL, and 197.6 ng/mL, respectively. However, at 12 h, C4-HSL increased from 512.2 ng/mL in the untreated group to 530.4 ng/mL in the AZM group (Fig. 2). With the addition of 2 μg/mL AZM, the HSL levels were noticeably lower than in the untreated controls, although there was no significant difference between the two groups (*P* > 0.05).

In the presence of AZM, a significant reduction in 3-oxo-C12-HSL was observed for PAO1. At 6 h, 8 h, 10 h, and 12 h, 3-oxo-C12-HSL levels were 156.2 ng/mL, 172.8 ng/mL, 89.6 ng/mL, and 140.5 ng/mL, respectively (*P* < 0.05) in the untreated group. In PAO1 treated with AZM, 3-oxo-C12-HSL levels were 58.3 ng/mL, 35.5 ng/mL, 17.6 ng/mL, and 49.4 ng/mL, respectively. The mutant strain PA-ΔrhlI also showed a reduction in 3-oxo-C12-HSL levels in the presence of AZM (*P* < 0.05) (Fig. 3).

### Effect of AZM on QS genes

**Figure 4:**
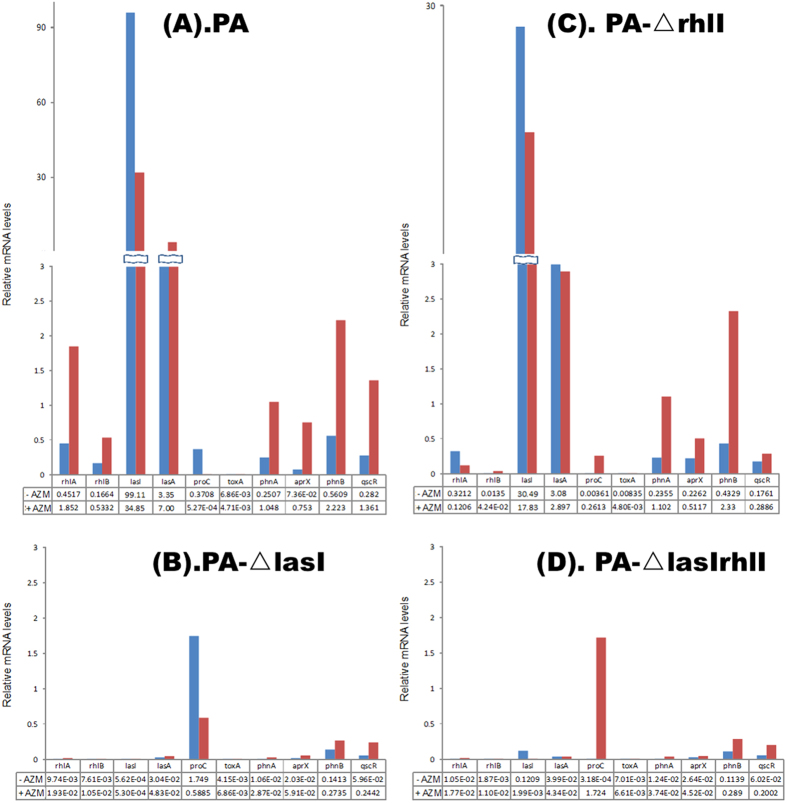
Comparison of gene expression with and without added 2 μg/mL azithromycin (AZM) in PAO1 (**a**) PA-Δ lasI (**b**) PA-Δ rhlI (**c**) and PA-Δ lasIrhlI (**d**). Series 1 (blue) denotes the untreated group and Series 2 (red) indicates the AZM group.

Real-time polymerase chain reaction (RT-PCR) was used to assess representative genes from the AHL and HAQ signaling systems. The experiment was conducted after 6 h of growth with or without 2 μg/ml AZM (Fig. 4). Selected genes regulated by lasR (aprX, toxA and lasA) and rhlR (rhlA and rhlB), genes of the HAQ system (phnA and phnB) and a repressor of lasI and rhlI, qscR, were assayed for expression in PAO1, PA-ΔrhlI, PA-ΔlasI and PA-ΔlasIrhlI. The expression of the genes is reported as a ratio of the target to a reference (rpoD).

The reduction in lasI expression in PAO1 was observed to correlate with the protein expression; 3-oxo-C12-HSL was reduced by 65% with AZM, matching the 79% reduction in protein levels under the same conditions. In the PAO1 strain, although lasI (3-oxo-C12-HSL) levels were reduced, as assessed by HPLC-MS/MS analysis, the expression of other QS genes was induced after addition of AZM. Additionally, the expression of other virulence genes, except toxA, showed a stronger repression in PAO1 compared to the mutants when AZM was not added. In the PA-ΔrhlI, PA-ΔlasI and PA-ΔlasIrhlI strains, the expression of virulence genes was induced with addition of AZM. Among the mutants, repression of virulence genes of the AHL system was stronger in PA-ΔlasI than in PA-ΔrhlI. Additionally, the expression of phnA and phnB from the HAQ system was greater in PA-ΔrhlI (and PAO1) compared to PA-ΔlasI.

## Discussion

In this study, we constructed *lasI* and *rhlI* single and double mutant strains, which had a complete deletion of the coding domain. A selective and rapid method for the simultaneous analysis of the two main HSLs in *PA* was also developed. Levels of 3-oxo-C12-HSL in PAO1 and PA-ΔrhlI showed a significant decrease following addition of AZM, while the accumulation of C4-HSL was not greatly influenced by AZM in the PAO1 strain. At the gene level, addition of AZM was observed to increase the expression of virulence genes in all backgrounds.

The las and rhl systems are hierarchically connected and regulate the timing and production of multiple virulence factors^37^. The las system positively regulates the expression of both rhlR and rhlI. When a strain is deficient in lasI, rhlI gene expression is repressed^38^. Because of this, no 3-oxo-C12-HSL was detected for PA-ΔlasI, and C4-HSL was below the detection limit. However, in PA-ΔrhlI, there was no C4-HSL detected, but 3-oxo-C12-HSL was produced normally. The lasI/rhlI gene mutation and AZM had no effect on *PA* growth.

In the early growth phase (2–4 h) of the PAO1 strain, there were no HSLs detected; 3-oxo-C12-HSL was first detected at 6 h. 3-Oxo-C12-HSL was first secreted in the exponential growth phase and was maintained until the early stationary phase. These results are in agreement with a previous report on PAO1, in which 3-oxo-C12-HSL at a concentration of 0.8 μmol/L was detected at 70.5 h^39^. Moreover, in this study, our detection limit was 0.05 ng/mL (0.17 nmol/L), more senstive than previous reports of 0.2 μmol/L^40^. Previous studies demonstrated that the expression of genes of the las system (lasR and lasI), which is affected by 3-oxo-C12-HSL, remains fairly constant throughout cultivation^41^. In contrast, the secretion of C4-HSL increased over time and reached a maximum at 12 h. In another study, the secretion of C4-HSL reached the maximum concentration at 42.5 h^42^.

At 2 μg/mL AZM, a concentration far below the MIC, the secretion of HSLs was inhibited through the blockade of QS. We observed that 2 μg/mL of AZM could decrease 3-oxo-C12-HSL secretion by nearly 79% and led to a noticeable reduction throughout the cultivation. The addition of 2 μg/mL AZM had a different effect on C4-HSL secretion, as it decreased C4-HSL secretion by 76% at t = 6 h and by 35% at t = 8~10 h, but secretion was not repressed at t = 12 h. Based on these results, we conclude that 2 μg/mL of AZM reduced 3-oxo-C12-HSL secretion during cultivation, but only repressed C4-HSL secretion in the early stages^43^. In this study, both an appropriate model system with a known time course involving autoinducers as well as AZM inhibition of QS signals in *PA* were clearly demonstrated. The resistance mechanisms of *PA* are very complex^44^,^17^, and HGT has been shown to be an important mechanism^41^. Conjugation, one of the most important mechanisms of HGT in the environment, involves the direct transfer of genetic material from cell to cell, usually in the form of plasmids or transposons^45^,^46^. It has been reported that HSLs have a positive effect on the conjugation of Ti plasmid transfer in *Agrobacterium tumefaciens*^42^. The basic model described here is intended to serve as a platform for further investigation into the effect of HSLs on conjugation in *PA* and inhibition of conjugation in *PA* by AZM.

**Figure 5:**
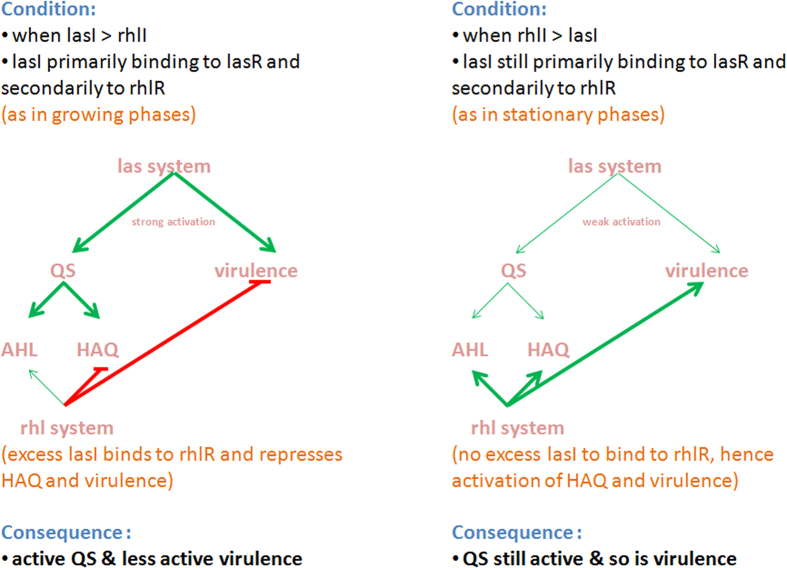
Model showing las and rhl system regulation of both QS and virulence. Green lines denote activation, and red denotes repression. The thickness of the lines indicates the strength of activation/repression.

Addition of AZM results in reduced HSLs at the protein level, while at gene level, AZM derepresses virulence genes. Transcriptional regulation of representative genes regulated by lasR and rhlR agreed broadly with previous findings. Stronger repression was observed in the wild-type strain compared to the mutants, and among the mutants, QS and virulence gene expression were observed only in the PA-ΔrhlI strain. Our results validated the hierarchy of las activation on the rhl system^47^. Without addition of AZM, all the virulence genes except toxA were repressed, with a stronger effect observed in the wild-type than in the mutants. This could be due to the phase in which the gene expression study was performed. toxA has been shown to be produced mainly during the early exponential phase of growth when grown aerobically^48^. As observed by Linares *et al*.^13^, sub-inhibitory concentrations of antibiotics could be the reason for the increased expression of QS and virulence genes (Fig. 4a). Another explanation for this could be that addition of AZM and the subsequent reduction in lasI expression (relative mRNA levels were 99.1 and 34.8, respectively, with AZM and without AZM) can be an inactivating signal, which has been previously shown to enhance virulence^49^,^32^. We hypothesize a novel regulatory circuit where lasI acts as an initial sensor of the optimal cell density required for activating virulence genes, while activated rhlI acts as a repressor of all the other QS systems (Fig. 5). In support of this idea, in the current study we observed that phnAB expression is reduced in PA-ΔlasI but not in PA-ΔrhlI (and PAO1). It has been shown previously that the las and rhl systems regulate quinolone signals, where lasI provides information to the cells about the QS activation, and rhlI suppresses the HAQ system. One of the regulators of las and rhl system is the quorum-sensing control repressor (qscR) protein, which functions as a las-rhl antagonist, and lasI is known to positively influence qscR. In the present study, low levels of qscR were observed in the absence of lasI and rhlI. Expression of qscR was 6 times higher in the wild-type compared to the mutant strains (Fig. 4), suggesting a feedback mechanism dependent on the concentration of lasI and rhlI via the regulation of qscR. The relationship between QS and virulence is complex. It is known that QS systems activate virulence and that AZM interferes with the QS activation by preventing virulence. Because AZM targets ribosomes, the effect of transcriptional regulation on QS can be inferred as a consequence of the reduction of the autoinducer.

The findings here provide basic data about the time course of AZM and the AZM effect on the synthesis of HSL. These results can further our understanding of antibiotic resistance and the interrelationship between QS and virulence. Here, we propose that QS and virulence can subsist individually, and regulated by the growth phase and the environment.

## Additional Information

**How to cite this article**: Zeng, J. *et al*. Mechanism of azithromycin inhibition of HSL synthesis in *Pseudomonas aeruginosa*. *Sci. Rep*. **6**, 24299; doi: 10.1038/srep24299 (2016).

## Supplementary Material

Supplementary Information

## Figures and Tables

**Table 1:** Strains and plasmids used in this study.

Strain or plasmid	Relevant genotype and/or phenotype	Source or reference
*PA* strains		
PAO1	Wild-type prototroph	This lab
PA-ΔJP1	Δ*lasI* derivative of PAO1, Tcr	ref. 1
PA-Δ*lasI*	Δ*lasI* derivative of PAO1	This study
PA-Δ*rhlI*	Δ*rhlI* derivative of PAO1	This study
PA-Δ*lasIrhlI*	Δ*lasI* derivative of PA-Δ*rhlI*	This study
*E. coli* strains		
DH5α	F-, Φ80dlacZΔM15,recA1,endA1,gvrA96,thi-1,hsdR17 (rK−, M+) supE44, relA1,deoR, Δ(lacZYA-argF)U169,Ap^S^,Km^S^	This lab
SM10λpir	*thi*1, *thr*1, *leu*B6, *sup*E44, *ton*A21, *lac*Y1, *recA*::RP4-2-Tc::Mu λ*pir* Km^R^	ref. 2
Plasmids		
pMD18T	ori (ColE1) Ap^r^	TaKaRa
pUC19	ori (ColE1) Ap^r^	TaKaRa
pCVD442	R6K ori, mobRP4, bla, sacB	ref. 3
pDS132	Derived from pCVD442, without IS1 sequences. bla gene replaced by the cat gene, C^R^	ref. 4
pMD18-mob	2.2 kb mob fragment from pCVD442 TA cloned into pMD18T, Ap^r^	This study
pMD18GM	0.83 kb aaCC1 fragment from pBBR-LuxAB cloned into pMD18T, GM^r^	This study
pBBR-LuxAB	luxAB, tra−, mob+, Gm^r^	ref. 5
pMD18AB	pMD18 with 1.1 kb upstream of *lasI* and 1 kb downstream of it, Ap^r^	This study
pUC19CD	pUC19 with 1.1 kb upstream of *rhlI* and 0.95 kb downstream of it, Ap^r^	This study
pG-Δ*lasI*	832 bp GM^r^ fragment from plasmid LuxAB cloned into pMD18BA, GM^r^, Ap^r^	This study
pGS-Δ*lasI*	1.8 kb sacB fragment from pCVD442 cloned into pG-Δ*lasI*, GM^r^, Ap^r^	This study
pGSM-Δ*lasI*	2.2 kb mob fragment from pCVD442 cloned into pGS-Δ*lasI*, GM^r^, Ap^r^	This study
pG-Δ*rhlI*	832 bp GM^r^ fragment from plasmid pBBR-LuxAB cloned into pUC19CD, GM^r^, Ap^r^	This study
pGS-Δ*rhlI*	1.8 kb sacB fragment from pCVD442 cloned into pG-Δ*rhlI*, GM^r^, Ap^r^	This study
pGSM-Δ*rhlI*	2.2 kb mob fragment from pCVD442 cloned into pGS-Δ*rhlI*, GM^r^, Ap^r^	This study

**Table 2:** Selected precursor and product ion m/z values, retention times and mass spectrometer parameters used for HSL analytes.

Analytes	Retention time (min)	Precursor ion (m/z)	Daughter ion (m/z)	Cone (v)	Collision Energy (v)
C4-HSL	5.09	172.1	102.1, 70.1	19	15
3-oxo-C12-HSL	7.0	298.2	102.1, 197.2	21	20

**Table 3:** The recovery test of HSLs from LB media.

Compound	Spiked level (μg/L)	Mean recovery (%)	RSD (%)
C4-HSL	10	64.4	3
	100	86.8	10
3-oxo-C12-HSL	10	97.6	11
	100	89.1	8
