# Supplementary material for: Mechanism of azithromycin inhibition of HSL synthesis in Pseudomonas aeruginosa
Source: Sci Rep. 2016 Apr 14;6:24299. doi: 10.1038/srep24299 (PMC4830939; doi:10.1038/srep24299)
Supplement: Supplementary Information [file srep24299-s1.pdf]

Mechanism of azithromycin inhibition of HSL synthesis in *Pseudomonas aeruginosa*

Jianming Zeng, Ni Zhang, Bin Huang, Renxin Cai, Binning Wu, Shunmei E, Chengcai Fang, Cha Chen

Appendix 1. Primers for RT-PCR

Name	Sense primer (5'-3')	Antisense primer (5'-3')
lasI	CGTGCTCAAGTGTTCAAGGA	AAAACCTGGGCTTCAGGAGT
lasA	CTGCTGGCTTTCAAGGTTTC	CCAGCAAGACGAAGAGGAAC
aprX	GCTGGGTAGCTACAACGAGG	AGACACCGATGTCCTTGACC
toxA	TGCTGCACTACTCCATGGTC	ACACCTTGATGTTCGAAGGC
rhlA	AGCTGGGACGAATACACCAC	GACTCCAGGTCGAGGAAATG
rhlB	GAGCGACGAACTGACCTACC	CGTACTTCTCGTGAGCGATG
qscR	AAACCGAGATGCTCAAGTGG	TGTTGCTGGAGTTGAGCTTG
phnA	CGTGTTCCAGATCGTACCGT	GACTCTCCGCGTCGTACTTC
phnB	CACTCGCTGGTGGTCAGTC	GTGAGAATCGACTCGGGATG
proC	GGCGTATTTCTTCCTGCTGA	TGGCCTGGAAGGATTTGAT
rpoD	CTGATCCAGGAAGGCAACAT	TGAGCTTGTTGATCGTCTCG
